# Progress of tumor-associated macrophages in the epithelial-mesenchymal transition of tumor

**DOI:** 10.3389/fonc.2022.911410

**Published:** 2022-07-28

**Authors:** Xiaoxiao Li, Ling Chen, Xiaobo Peng, Xianbao Zhan

**Affiliations:** Department of Oncology, Changhai Hospital, Naval Military Medical University, Shanghai, China

**Keywords:** tumor metastasis, tumor-associated macrophages, epithelial-mesenchymal transformation, tumor microenvironment, treatment

## Abstract

As a significant public health problem with high morbidity and mortality worldwide, tumor is one of the major diseases endangering human life. Moreover, metastasis is the most important contributor to the death of tumor patients. Epithelial-mesenchymal transition (EMT) is an essential biological process in developing primary tumors to metastasis. It underlies tumor progression and metastasis by inducing a series of alterations in tumor cells that confer the ability to move and migrate. Tumor-associated macrophages (TAMs) are one of the primary infiltrating immune cells in the tumor microenvironment, and they play an indispensable role in the EMT process of tumor cells by interacting with tumor cells. With the increasing clarity of the relationship between TAMs and EMT and tumor metastasis, targeting TAMs and EMT processes is emerging as a promising target for developing new cancer therapies. Therefore, this paper reviews the recent research progress of tumor-associated macrophages in tumor epithelial-mesenchymal transition and briefly discusses the current anti-tumor therapies targeting TAMs and EMT processes.

## Introduction

**Figure 1:**
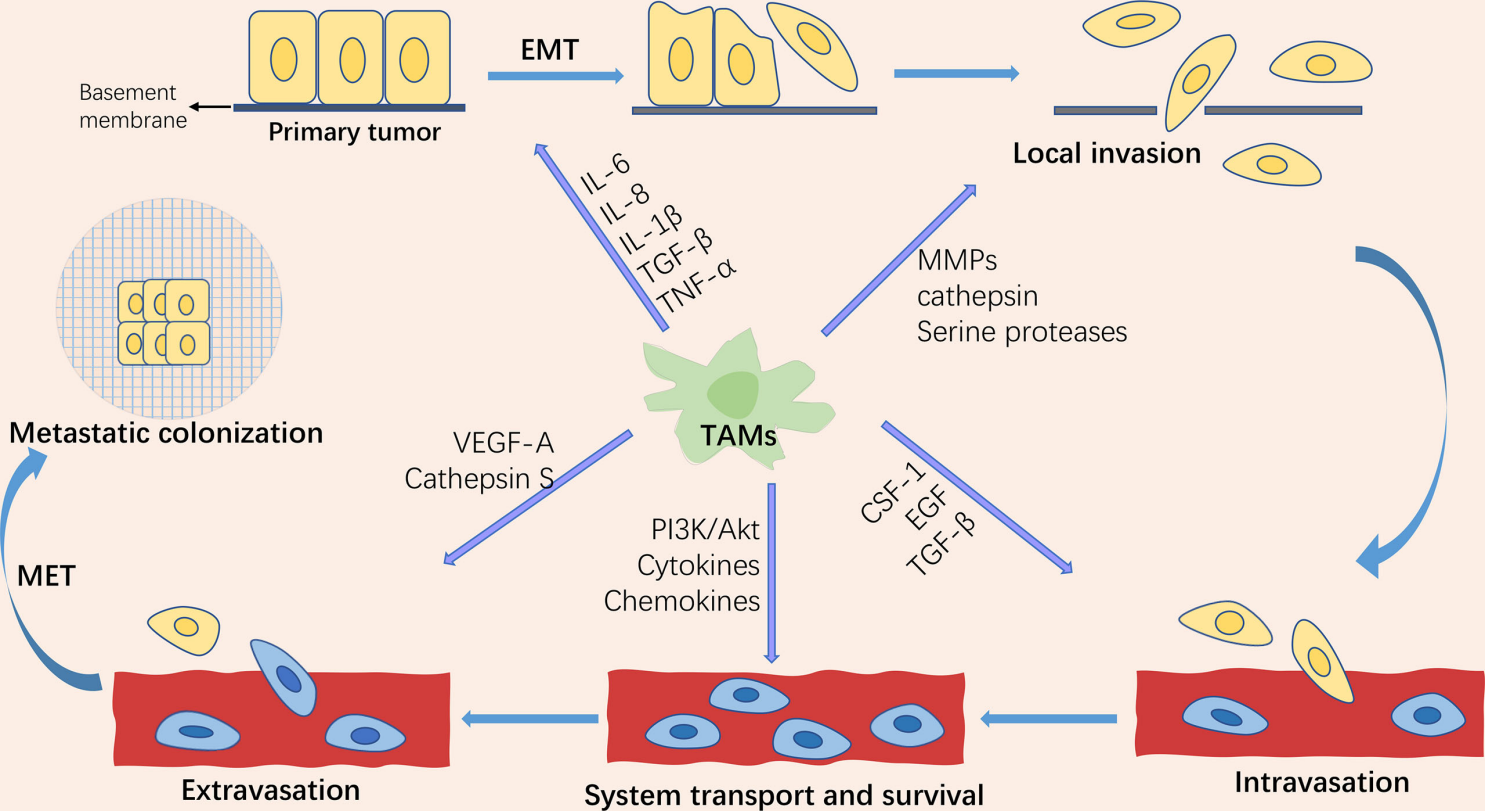
Schematic representation of the tumor invasion-metastasis cascade and the role of tumor-associated macrophages (TAMs) in tumor metastasis. The tumor invasion-metastasis cascade includes tumor cell EMT, invasion of the surrounding extracellular matrix and stromal cell layer, intravasation, survival and transport in the circulatory system, extravasation, survival in the distant tissue microenvironment, and colonization and growth at the metastatic site. TAMs are involved in regulating almost every step of the tumor invasion-metastasis cascade through the secretion of multiple factors.

Tumor metastasis is the leading cause of cancer-related death, accounting for approximately 90% of deaths, yet the process is still poorly understood (1). Metastasis is a dynamic, multi-step, and complicated process. The invasion-metastasis cascades consist of the following steps: (1) Epithelial-mesenchymal transition (EMT) of cancer cells; (2) Invasion of the surrounding extracellular matrix (ECM) and stromal cell layer; (3) Intravasation of tumor cells into the vascular lumen; (4) Transport through the circulatory system; (5) Extravasation in distant tissues and organ parenchyma; (6) Survival in the microenvironment of distant tissues; (7) Colonization and growth at metastatic sites (2, 3)(**Figure 1**). As one of the hallmarks of cancer (4), metastasis is driven not only by intrinsic changes in tumor cells but also by interactions between tumor cells and the components of the microenvironment in which they reside, known as the “seed and soil” doctrine. This theory was first proposed by Stephen Paget in 1889 and is now widely accepted as a critical theory related to metastasis (5). The components that make up the “soil” are termed the tumor microenvironment (TME). Although widely studied, only part of it is understood because TME is highly intricate. Currently, known components include tumor cells, fibroblasts, inflammatory mediators, immune cells, reactive oxygen species, and tumor-associated cytokines.

Tumor-associated macrophages (TAMs) are one of the major infiltrating immune cells in the tumor microenvironment. Studies have revealed that TAMs orchestrate almost all of the above cascade steps of tumor metastasis (6). EMT is a vital biological process in the progression of primary tumors to metastasis. EMT, as the primary central part of tumor metastasis, endows tumor cells with the ability of migration, invasion, anti-nesting apoptosis (7), evading immune surveillance (8), and chemotherapy resistance (9). EMT is a critical step in the acquisition of tumor cell migration and invasion capabilities (10). TAMs play a crucial role in regulating tumor cell proliferation, extracellular matrix remodeling, tumor cell invasion and metastasis, lymphangiogenesis, and angiogenesis by producing various cytokines, growth factors, chemokines, protein hydrolases, and inhibitory immune checkpoint proteins. In this review, we present the progress of TAM in tumor epithelial-mesenchymal transformation in recent years.

## EMT and tumor metastasis

Epithelial-mesenchymal transition (EMT) refers to a series of biological processes in which polarized epithelial cells attached to the basement membrane undergo a variety of biochemical and molecular changes and transform into mesenchymal-like cells. As a physiological process, EMT performs an important role in organogenesis, tissue development, and wound healing (11). Also, pathologically, EMT is the basis of tumor progression and metastasis, which endows tumor cells with the ability of migration, invasion, anti-nesting apoptosis, and chemotherapy resistance (12). Studies have shown that EMT is also one of the most important biological processes in inducing stem cell properties, meaning that non-cancer stem cells can be induced into a cancer stem cell (CSC)-like state (13). However, some studies have questioned the necessity of EMT in tumor metastasis (14). Zheng et al. (15)reported that tumor cells can metastasize without activating EMT in genetically engineered mice with pancreatic cancer and mice with spontaneous metastasis of breast cancer. Similarly, Fischer et al. (16)showed that EMT is not necessary for lung metastasis. Nevertheless, these few findings have been challenged by other researchers, and it is still widely accepted that EMT is needed in the process of tumor metastasis. EMT is divided into three types: type 1 EMT is involved in embryonic implantation, embryogenesis, and organ development; type 2 EMT is related to the processes of wound healing, tissue regeneration, and organ fibrosis; and type 3 EMT is associated with cancer progression and metastasis (17).

**Figure 2:**
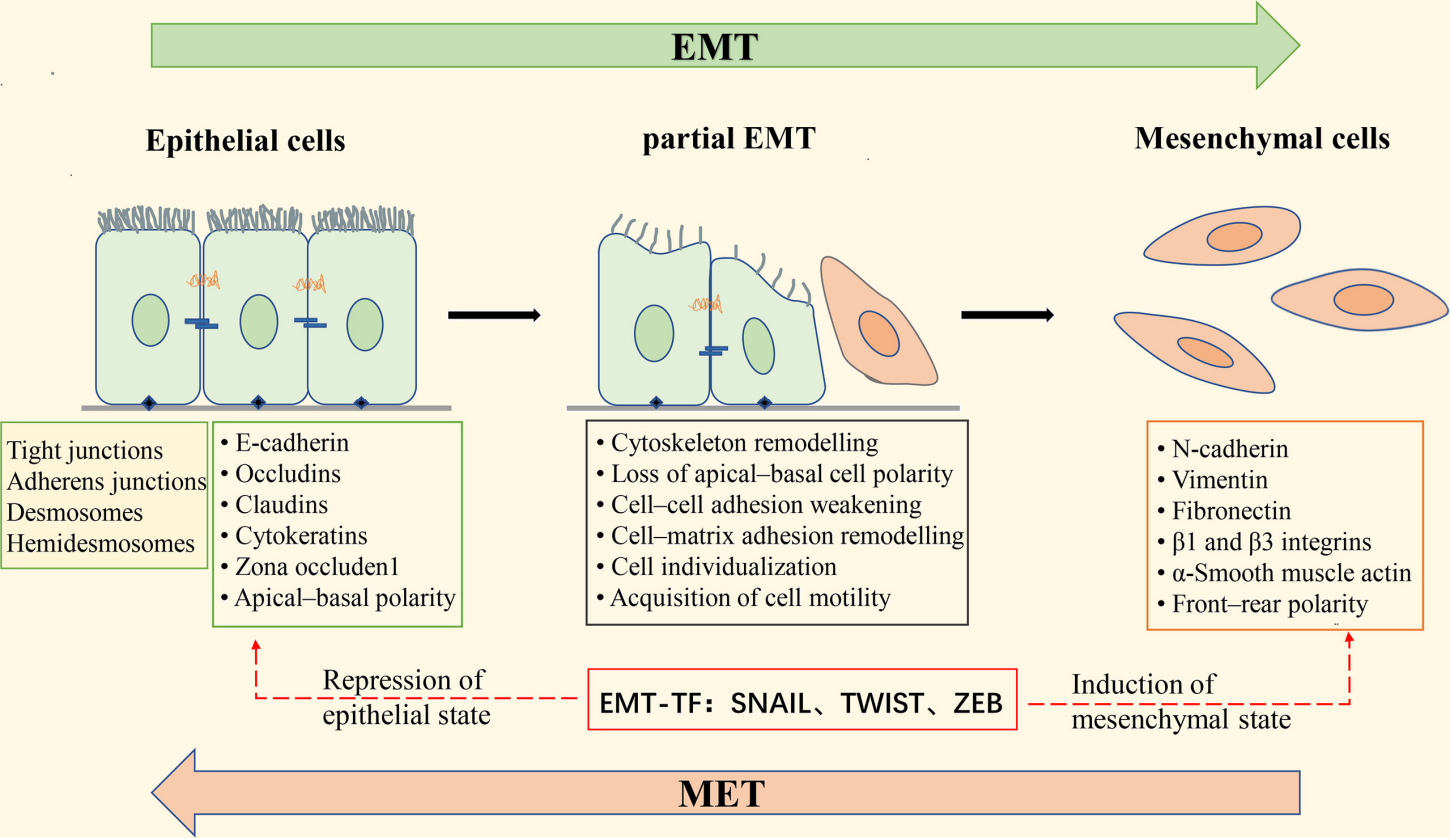
Schematic diagram of epithelial-mesenchymal transformation. Apical-basal polar epithelial cells are attached by tight junctions, gap junctions, desmosome, and adhesion junctions, and to the basement membrane by Hemidesmosome; epithelial cells express epithelial cell markers such as E-cadherin, Occludins, Claudins, and Cytokeratins. Induction of epithelial-mesenchymal transition (EMT) leads to the expression of the EMT transcription factors (EMT-TF) ZeB, Snail, and Twist, which repress the expression of epithelial cell-associated genes and simultaneously activate the expression of mesenchymal cell-associated genes. These changes in gene expression result in morphological alterations of epithelial cells, deconstruction of intercellular junctions, the acquisition of front-rear polarity, and the ability of cells to become motile and invasive. During the transition from epithelial to mesenchymal cells, the cells undergo a “partial EMT” state, in which the cells are intermediate between a fully epithelial and fully mesenchymal state, retaining both epithelial and mesenchymal characteristics. EMT is reversible, and the process of mesenchymal-epithelial transformation (MET) allows the cells that have undergone EMT to return to the epithelial state.

During EMT, the epithelial cytoskeleton is reorganized, and the cell morphology is changed from squamous, cuboidal, or columnar to a spindle-shaped and elongated shape; epithelial cell-cell adhesion is reduced, and intercellular junctions are deconstructed, including tight junctions, adherent junctions, and gap junctions; the Crumbs, PAR, and Scribble (SCRIB) polarity complexes are disrupted, resulting in the loss of apical-basal polarity in epithelial cells (18). At the same time, epithelial cell markers such as E-cadherin, β-catenin, Desmosomes, and cytokeratin are decreased, while mesenchymal cell markers such as N-cadherin, fibronectin, vimentin, and α-smooth muscle actin (α-SMA) are increased; actin is remodeled so that cells can elongate dynamically and move directionally (19). These phenotypic changes are driven by alterations in the signaling pathways that activate EMT-related transcription factors. EMT-inducible transcription factors (EMT-TFs) mainly include the zinc finger E-box-binding transcription factors ZEB1 and ZEB2, SNAIL (also known as SNAI1), SLUG (also known as SNAI2), and the helix-loop-helix (HLH) transcription factors TWIST1 and TWIST2. Activation of these transcription factors can repress the expression of epithelial cell-associated genes and promote the expression of mesenchymal cell-associated genes (**Figure 2**). Extracellular signals that induce EMT in tumor cells, such as tumor necrosis factor-α (TNF-α), transforming growth factor-β (TGF-β), hepatocyte growth factor (HGF), epidermal growth factor (EGF), fibroblast growth factor (FGF), mitogenic growth factor (MGF), vascular endothelial growth factor (VEGF), IL-6, IL-1β, Etc., which act on EMT-related transcription factors through activation of TGF-β, Wnt-β-catenin, Notch, Hedgehog, PI3K-AKT, NF-κB, MAPK, and other signaling pathways, leading to down-regulation of epithelial cell markers and up-regulation of mesenchymal cell markers (18).

EMT has long been considered a binary process consisting of two distinct cell populations, epithelial and mesenchymal (20); that is, cancer cells undergoing EMT completely lose epithelial phenotypes and fully acquire mesenchymal phenotypes. However, there is growing evidence that this is not the case. It has been found that the transition from the epithelial state to the mesenchymal state of cancer cells with EMT is usually incomplete, resulting in cells in the intermediate state retaining the characteristics of both epithelial and mesenchymal cells, which is called partial, incomplete, or mixed EMT state (21–23). “Partial EMT” (pEMT) has been widely observed in a variety of cancers, such as lung cancer (24), colorectal cancer (25), and prostate cancer (26). Recent studies have found that cells in the pEMT state exhibit higher tumor metastatic potential, stem cell properties, and drug resistance than epithelial or mesenchymal cells alone (27, 28).

## Tumor-associated macrophages (TAMs)

As a type of intrinsic immune cell, macrophages perform a wide variety of functions, including regulating tissue homeostasis, defending against pathogens, and promoting wound healing. Macrophage monitoring is critical to prevent tumor growth, and there is evidence that activated macrophages can recognize and kill transformed and mutated cells in humans (29). Initially, it was thought that macrophages were mainly derived from circulating monocytes of bone marrow hematopoietic stem cell origin (30). However, a large body of evidence suggests that the majority of tissue-resident macrophages originate from yolk sac progenitors, such as alveolar macrophages, brain macrophages, Kupffer cells, abdominal macrophages, epidermal Langerhans cells, and brain microglia (6). According to the function of macrophages, macrophages are divided into classically activated M1 macrophages and alternatively activated M2 macrophages. M1 macrophages secrete pro-inflammatory cytokines such as IL-12, tumor necrosis factor (TNF)-α, CXCL-10, and interferon (IFN)-γ, and produce high levels of nitric oxide synthase (NOS), which promote inflammatory response, pathogen clearance, and anti-tumor immunity. In contrast, M2 macrophages secrete anti-inflammatory cytokines such as IL-10, IL-13, IL-4, arginase-1, mannose receptor (MR, CD206), and scavenger receptor, which tend to exert an immunosuppressive phenotype that benefits tissue repair and tumor progression (31). In tumor microenvironment (TME), CD163 and CD206 are commonly used to identify M2 macrophages, while CD86 is a standard M1 marker. The biological process of switching between M1 (anti-tumorigenic) and M2 (pro-tumorigenic) is called “macrophage polarization”. Macrophage polarization is regulated by many microenvironmental cytokines, chemokines, growth factors, and other signals from tumor and stromal cells. Macrophages infiltrating in tumor tissue or aggregating in the solid tumor microenvironment are defined as tumor-associated macrophages (TAMs), which are one of the central infiltrating immune cells in the tumor microenvironment.

It is commonly accepted that tumor-associated macrophages (TAMs) are highly similar to M2-polarized macrophages. In the past few decades, many studies have shown a significant positive correlation between the number and density of TAMs infiltration and poor patient prognosis in most tumor types (32, 33). One of the mechanisms of TAMs enhancing cancer cell invasion involves the paracrine loop, in which macrophages and tumor cells interact with each other. The colony-stimulating factor-1 (CSF-1) produced by tumor cells binds to CSF-1R on the surface of macrophages, thus promoting macrophage proliferation, migration, and polarization to the M2-like phenotype (34). Meanwhile, macrophages release epidermal growth factor (EGF), which enhances tumor cell invasion and migration, and further stimulates tumor cells to secrete CSF-1, thus forming a positive feedback loop between tumor cells and macrophages (35). TAMs in the tumor microenvironment promote the immunosuppressive tumor microenvironment by expressing chemokines and cytokines (36). For example, chemokines CCL5, CCL22, and CCL20 secreted by TAMs can recruit regulatory T(Treg) cells. TAMs can also inhibit the antitumor activity of infiltrated NK cells and T cells and promote the immunosuppressive tumor microenvironment combined with myeloid suppressor cells (MDSCs), tumor-associated dendritic cells, and neutrophils (37). In addition, M2 macrophages can also promote the migration of tumor cells and tumor stromal cells by secreting matrix metalloproteinases (MMPs), serine proteases, and cathepsin that disrupt the basement membrane and extracellular matrix (38). Several studies have provided strong evidence that the presence of TAM during metastatic extravasation is critical for the successful formation of metastatic foci. Qian et al. (39)demonstrated in an animal model of breast cancer metastasis, using an intact lung imaging system, that macrophages are required for tumor cell extravasation; more importantly, ablation of this macrophage population resulted in a significant decrease in the rate of tumor cell extravasation, thus inhibiting the growth of metastatic tumors. The same conclusion was obtained by Penny et al. in pancreatic ductal adenocarcinoma (PDAC), where TAM enhanced the extravasation of tumor cells from blood vessels and induced higher levels of EMT compared to steady-state macrophages (40). Mechanistically, it has been shown that at metastatic sites, tumor cell-derived CCL2 recruits inflammatory monocytes to metastatic sites, where they differentiate into metastasis-associated macrophages that produce vascular endothelial growth factor-A and cathepsin S, thereby promoting cancer cell extravasation (41, 42).

The tumor metastatic microenvironment (TMEM) is an anatomical structure composed of three parts: TAMs, tumor cells, and endothelial cells, and identifiable on tissue sections. TMEM is a predictor of increased hematogenous metastasis and poor prognosis and can predict the metastatic ability of breast cancer (43). Tumor hematogenous metastasis is the main pathway of malignant tumor metastasis. When the solid tumor grows to a specific size, a process called “angiogenesis switch” will be initiated by various mechanisms to trigger the high-density vascular system to provide nutrition and waste removal for tumor cells (44). In addition to contributing to the establishment of the immunosuppressive microenvironment and the degradation of the extracellular matrix, TAMs also support tumor growth by inducing angiogenesis. There is an increasing number of evidence that TAMs play an essential role in regulating the “angiogenic switch” by secreting various pro-angiogenic mediators, such as vascular endothelial growth factor A (VEGF-A), thymidine phosphorylase, urokinase-type fibrinogen activator (uPA), and adrenomedullin (ADM) (45, 46). It was found that macrophages express ligands for two inhibitory receptors, programmed cell death protein 1 (PD-1) and cytotoxic T lymphocyte antigen 4 (CTLA-4). In areas of tumor hypoxia, hypoxia can inhibit intratumor cytotoxic T cell responses by upregulating the expression of PD-L1 on the surface of TAMs *via* hypoxia-inducible factor 1-α (HIF-1α) (47).

## The role of TAMs in epithelial-mesenchymal transition of tumors

**Figure 3:**
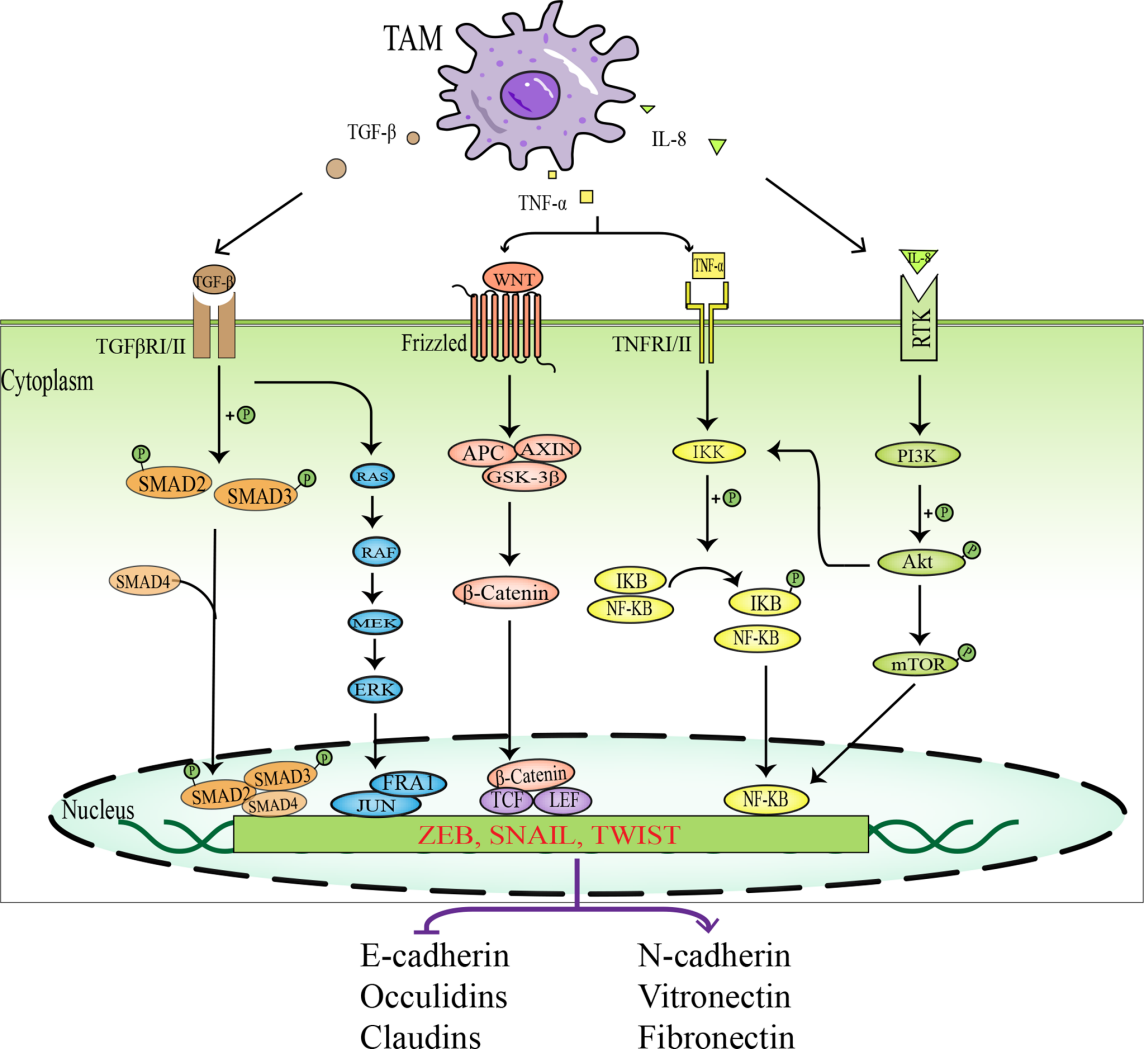
Schematic diagram of signal pathways in which TAMs promotes EMT in tumor cells. TAMs activate EMT- IF by secreting various factors, such as TGF-β, TNF-α, and IL-8, which act on intracellular related signaling pathways, including TGF-β- SMAD signaling pathway, MAPK signaling pathway, WNT-β-Catenin signaling pathway, NF-KB signaling pathway, and PI3K-AKT signaling pathway. EMT-IF can inhibit the expression of epithelial cell markers and promote the expression of mesenchymal cell markers, which leads to EMT in tumor cells.

Apart from accumulating intrinsic changes within the malignant tumor cells, the tumor microenvironment also provides a fertile ground for tumor progression and metastasis. The “dialogue” between tumor cells and various stromal cells in the tumor microenvironment is of great importance in tumor progression and metastasis (48). The interaction between tumor cells and TAMs plays a pivotal role in the epithelial-mesenchymal transition (EMT) process. TAMs induce EMT in tumor cells by secreting a host of cytokines and growth factors, such as transforming growth factor-beta (TGF-β), tumor necrosis factor-α (TNF-α), interleukin-6 (IL-6), and interleukin-8 (IL-8), thereby promoting tumor invasion and metastasis. The relevant intracellular signal pathways involved in the promotion of EMT in tumor cells by TAMs are shown in **Figure 3**.

### TGF-β

The TGF-β family is a group of extracellular growth factors, including TGF-βs, activins, and bone morphogenetic proteins (BMPs), which are involved in regulating tumor growth, migration, angiogenesis, and immune response (49). TGF-β has dual effects on tumor behavior. In the early stages of tumorigenesis, TGF-β, as a potent growth inhibitor, can inhibit the epithelial cell cycle and promote apoptosis, thus suppressing tumorigenesis and progression (50). In the middle and late stages of the tumor, however, TGF-β becomes a “catalyst” for cancer progression, inducing and promoting epithelial-mesenchymal transition (EMT), enhancing the invasiveness of tumor cells, and participating in the malignant progression of tumors (51, 52). Some studies have provided reasonable explanations for the above phenomenon. In the early stages, TGF-β is involved in tumor cell inhibition and apoptosis as the main tumor suppressor. In the middle and late stages of tumors, tumor cells become resistant to TGF-β or are reprogrammed by tumor cells to promote tumor growth (53).

In the tumor microenvironment, TGF-β1 is generated by tumor cells and infiltrating immune cells, such as TAMs, myeloid-derived suppressor cells (MDSC), and regulatory T cells (Treg). TGF-β1 can induce EMT in tumor cells, promote tumor cells to evade immune surveillance, and facilitate tumor spread and metastasis (54). By binding to a complex of transforming growth factor-beta receptor 1 (TGFβR1) and transforming growth factor-beta receptor 2 (TGFβR2), TGFβ1 leads to phosphorylation of SMAD2 and SMAD3 and combines with SMAD4 to form a SMAD trimeric complex, which translocates to the nucleus and acts as a transcription factor to regulate the expression of EMT-related genes.

The TGF-β1-induced SMAD complex activates the expression of mesenchymal genes, such as vimentin and fibronectin, as well as EMT transcription factors, and represses the expression of the epithelial gene E- cadherin. Simultaneously, EMT-TF can upregulate the expression of the TGF-β ligand, thus establishing a positive feedback loop between EMT-TF and TGF-β pathway, which is beneficial for cells to maintain the EMT state (18). Cheng et al. (55)found that pancreatic ductal adenocarcinoma (PDAC) cells incubated with TAMs or TAM cell-conditioned cultures (TAM-CM) showed higher migration and invasion rates than controls, and further demonstrated that TAMs induced EMT in PDAC cells *via* the TGF-β-Smad2/3/4-Snail signaling axis, leading to tumor cell migration. In contrast, the tumor-promoting effect of TAMs was eliminated after the application of TGF-β signaling pathway inhibitors and neutralizing TGF-β antibodies. These results suggest that TAMs promote PDAC progression through the TGF-β signaling pathway. In addition, the TGF-β/SMAD signaling pathway also “cooperates” with other non-SMAD signaling pathways, such as ERK, p38MAPK, PI3K-AKT, and Rho-like GTPases, to participate in the regulation of EMT (56). TGF-β can also induce EMT by regulating microRNA (microRNA) and long-stranded non-coding RNA (LncRNA). TGF-β can also induce EMT by regulating microRNA and long-stranded non-coding RNA (LncRNA) (57). Moreover, TGF-β1 can promote tumor invasion and metastasis by affecting the tumor microenvironment, for example, through immunosuppression and angiogenesis (58).

### TNF-α

The tumor necrosis factor (TNF) family is an important class of cytokines, which plays a role in regulating a range of physiological and pathological responses, including cell proliferation, differentiation, apoptosis, immune response, and inflammation. As a critical pro-inflammatory cytokine in the tumor microenvironment, TNF-α is mainly secreted by macrophages and tumor cells. Initially, exogenous TNF-α was observed to cause hemorrhagic necrosis of tumors and promote tumor vascular destruction, so it was considered one of the anti-cancer cytokines that could inhibit tumor progression (59, 60). Follow-up studies have found that endogenous TNF-α derived from tumor cells and macrophages at the tumor site does not have antitumor activity but rather promotes tumor growth and metastasis (61, 62).

TNF-α belongs to the cytokine TNF/TNFR superfamily and is a type II transmembrane protein. It can act as a membrane-integrated protein or as a soluble molecule to induce EMT by interacting with its two specific receptors, TNFR1 and TNFR2, to initiate different signaling pathways. TNF-α can induce EMT in tumor cells by inhibiting the expression of epithelial marker E-cadherin, upregulating the expression of mesenchymal markers such as vimentin, N-cadherin, and fibronectin, and activating matrix metalloproteinase-9 (MMP-9), thus enhancing tumor invasion and migration activity (60). A study showed that TNF-α derived from M2TAMs promotes EMT and tumor stem cell properties in hepatocellular carcinoma cells *via* the Wnt/β-catenin pathway (63). Furthermore, TNF-α and TGF-β released from macrophages interact during EMT, jointly regulating tumor invasion and metastasis (64). For example, EMT in breast cancer cells is regulated by the TGF-β/SMAD pathway and activated by TNF-α/NF-kB/Twist, both of which synergistically promote breast cancer cell migration and metastasis (65, 66). Likewise, in cervical cancer, TNF-α and TGF-β collaboratively induce EMT and tumor stem cell-like properties through the NF-kB/Twist axis (67). Song et al. (68) found that in colorectal cancer cell lines, TNF-α and TGF-β induced EMT-like changes through NLRP3/Snail1 axis-dependent manner or by increasing the expression of claudin-1. At the same time, TNF-α and TGF-β are also antagonistic under some microenvironmental conditions. It has been reported that in hepatocellular carcinoma, TGF-β treatment upregulates autophagy gene expression, which strongly activates autophagy and induces EMT. However, TNF-α suppresses TGF-β-induced EMT levels by inhibiting autophagy (69).

### Other cytokines inducing EMT

Several studies have found that mesenchymal cancer cells promote macrophage activation to a TAM-like phenotype by secreting GM-CSF. In turn, TAMs can induce EMT in neighboring epithelial cancer cells by producing CCL-18, thus forming a positive feedback loop between TAMs and EMT (70). Interleukin-8 (IL-8 or CXCL8), a granulocyte chemokine, has multiple functions in the tumor microenvironment (TME), such as recruitment of immunosuppressive cells, promotion of tumor angiogenesis, and EMT (71). Deng et al. (72)demonstrated that IL-8 overexpression promotes EMT and migration of triple-negative breast cancer cells (TNBCs) through PI3K-Akt signaling pathway and E-cadherin downregulation. In ovarian cancer, IL-8 mediates tumor cell EMT *via* the Wnt/β-catenin pathway to promote ovarian cancer metastasis (73).

Studies have indicated that the concentration of macrophage-derived IL-10 is almost ten times higher than that of leukocyte-derived IL-10 in tumors (74). IL-10 is involved in the induction of EMT in tumor cells. A study proved that when M2-type TAMs were co-cultured with pancreatic cancer cell lines PANC-1 and BxPC-3, it increased the fibroblast morphology of pancreatic cancer cells, with reduced expression of the epithelial marker E-cadherin and increased expression of the mesenchymal markers Vimentin and Snail. In contrast, the application of RNAi technology to interfere with TLR4 or anti-TLR4 and IL-10 neutralizing antibodies significantly inhibited the downregulation of E-cadherin and reduced the upregulation of Vimentin and Snail, suggesting that TAMs promote EMT in pancreatic cancer cells partly through TLR4/IL-10 signaling (75).

Interleukin-6 (IL-6), another inflammatory cytokine secreted by TAM, is upregulated in most common human tumors. Elevated serum levels of IL-6 indicate a poorer prognosis for tumor patients (76). Research revealed that in the interaction between macrophages and lung cancer cells, IL-6 promotes the translocation of β-catenin from the cytoplasm to the nucleus *via* the COX-2/PGE2 pathway, which induces EMT and promotes tumor cell invasion (77). Since IL-6 concentration in the serum of patients is associated with advanced tumor stage and overall survival, it may be used as a biomarker for preoperative assessment of prognosis.

M2-polarized TAMs regulate the microenvironment by increasing the secretion of cytokines and chemokines and activating the AKT3/PRAS40 signaling pathway to promote EMT in intrahepatic cholangiocarcinoma cells (78). Zhang et al. (79)have indicated that a HIF-1α/IL-1β signaling loop exists between cancer cells and tumor-associated macrophages in a hypoxic microenvironment, which leads to EMT in hepatocellular carcinoma cells.

## Antitumor therapy targeting TAMs and EMT

In recent years, with intensive study of TAMs and EMT in tumor metastasis, the role of TAMs in tumor development has received increasing attention. TAMs are considered as a potential biomarker for cancer diagnosis and prognosis, as well as a potential target for cancer therapy. At present, many therapeutic strategies have been developed for targeting TAMs and EMT processes, as shown in **Table 1**.

**Table 1:** Antitumor therapy targeting TAMs and EMT.

Study sponsor	Tumor type	Mechanism	Approach	Reference
**Targeting TAMs**				
Gomez-Roca et al.	Metastatic solid tumors	Blocking the CSF-1/CSF1R axis	Reduces the number of TAMs in tumors	(80)
Zhu et al.	Hepatocellular carcinoma	Blocking OPN/CSF1/CSF1R axis	Prevents TAMs trafficking and sensitizes HCC to anti-PD-L1 blockade	(81)
Hui et al.	Esophageal squamous cell carcinoma	Blocking the CCL2-CCR2 axis	Prevents the recruitment of TAMs and enhances the antitumor effect of CD8+ T cells	(82)
Jaynes et al.	Solid tumors	Induces of conformational conversion of mannose receptor CD206 expressed on M2 TAMs	Reprograms M2-like TAMs to anti-tumor M1-like TAM phenotype	(83)
Giurisato et al.	Solid tumors	Blocks ERK5 to suppress STAT-induced gene expression	Reprograms macrophages toward an antitumor state	(84)
Kuo et al.	Solid tumors	Blocking the CD47/SIRPα signaling pathway	Promotes phagocytosis of macrophages to tumor cells and enhances immune response	(85)
Barkal et al.	Ovarian cancer and breast cancer	Blocking the CD24/Siglec-10	Increases phagocytosis of macrophages to tumor cells	(86)
**Targeting EMT**				
Zhu et al.	Lung adenocarcinoma	Inhibiting ZEB2	Targets EMT-inducible transcription factors	(87)
Castaneda et al.	Solid tumors	Degrading FOXC2 and inducing cadherin switch	Reverses EMT process and inhibits tumor metastasis	(88)
Herbertz et al.	Solid tumors	Inhibiting the activation of the typical TGF-β pathway	Targets EMT-related pathways	(89)
Liu et al.	Breast cancer	Targeting the EMT inducer CD146	Reverses EMT process	(90)
Jonckheere et al.	Solid tumors	Inhibiting LSD1	Inhibits EMT process	(91)

CSF-1, colony-stimulating factor-1; OPN, osteopontin; HCC, Hepatocellular carcinoma; ERK5, extracellular-regulated protein kinase 5; SIRPα, Signal regulatory protein α; Siglec-10, sialic acid-binding Ig-like lectin 10; FOXC2, Fork head box protein C2; LSD1, Lysine-specific histone demethylase 1.

### Targeting TAMs

Given the tumor-promoting effects of TAMs, a multitude of strategies have been devised to counteract the impact of TAMs. Generally speaking, these strategies can be classified into two categories: reducing the number of TAMs in the tumor or altering the function of TAMs in the TME.

Limiting the number of TAMs in the tumor can be achieved by eliminating existing TAMs and inhibiting TAMs recruitment. The most established approach to reduce TAM viability is currently by blocking the CSF-1 (also known as MCSF)/CSF-1R axis, as colony-stimulating factor-1 (CSF-1) is considered one of the most important recruitment factors for macrophages and TAM polarization factors (92). This method not only reduces the source of TAMs by blocking monocyte differentiation but also reduces the survival rate of existing TAMs. Therefore, several inhibitors targeting the CSF-1/CSF1R axis have been developed and are being studied in clinical trials as monotherapy or in combination with chemotherapeutic agents. For example, Gomez-Roca et al. (80)found that Emactuzumab alone or coupled with paclitaxel for advanced or metastatic solid tumors significantly reduced the number of TAMs in tumors. Also, it was shown that blockade of CSF1/CSF1R increased the sensitivity of tumors to other immunotherapies, such as anti-PD-L1 antibody therapy (81). Because of the role of chemokine CCL2 in the recruitment of circulating monocytes to tumors, much work has been done to inhibit the CCL2/CCR2 axis (93). Hui et al. (82)found that blocking the CCL2-CCR2 axis can significantly reduce tumorigenesis by preventing the recruitment of TAMs and enhancing the antitumor effect of CD8+ T cells in the tumor microenvironment. However, removal of CCL2 blockade treatment resulted in the resumption of tumor progression since TAMs have recruited to the tumor site again (94).

As M1 and M2 macrophages are highly plastic and can transform into each other in response to changes in the tumor microenvironment or therapeutic intervention, “reprogramming” TAMs into an anti-tumor phenotype is a promising therapeutic strategy. Anti-tumor macrophages (M1 type) have the ability to eliminate and destroy tumor cells. Toll-like receptors (TLR) are a family of receptors involved in innate immunity, which can alter the phenotype of macrophages. These pattern recognition receptors can react to bacterial particles or bacterial and viral genomes (e.g., DNA or RNA) to trigger the release of pro-inflammatory cytokines (95). Studies have shown that TLRs located in cells (TLR3, TLR7, TLR8, or TLR9) are more effective in triggering anti-tumor immune responses than extracellular TLRs (TLR1, TLR2, TLR4, or TLR6) (96). Therefore, several studies have focused on assessing the ability of intracellular TLR agonists to induce reprogramming of TAMs. At present, there have been some successes in this field, such as Imiquimod (TLR7 agonist), which has passed Phase III clinical trials and received FDA approval for the treatment of squamous and basal cell carcinomas (97). RP-182 is a synthetic peptide analog that selectively induces conformational transitions of the mannose receptor CD206 expressed on M2-type TAMs, thus reprogramming M2-like TAMs to an anti-tumor M1-like TAM phenotype (83). It has been found that ERK5 plays a role in determining macrophage polarity, and the growth of transplanted tumors in ERK5 gene-deficient mice is inhibited. Further studies have confirmed that STAT3 activation *via* phosphorylation of Tyr705 is reduced in ERK5-deficient TAMs. Thus, inhibiting STAT3-induced gene expression by blocking ERK5 may be a strategy to treat cancer by reprogramming macrophages into an anti-tumor state (84). The phagocytic function of macrophages is regulated by the inhibitory receptor signaling regulatory protein α (SIRPα) expressed on macrophages, whose ligand is CD47, a “do not eat me” signal, overexpressed on tumor cells. The CD47/SIRPα axis is the predominant mechanism by which tumor cells resist macrophage phagocytosis (98). Kuo T C et al. (85)found that blocking the CD47/SIRPα signaling pathway effectively promoted phagocytosis of macrophages against tumor cells and enhanced innate and adaptive immune responses to promote anti-tumor activity. Furthermore, in many tumor types, CD24 is an anti-phagocytic signal expressed on the surface of tumor cells. It helps tumor cells to avoid macrophage attack by interacting with inhibitory sialic acid-binding Ig-like lectin 10 (Siglec-10) expressed by TAMs (99). Barkal et al. (86) found that blocking the CD24-Siglec10 interaction with anti-CD24 monoclonal antibody significantly increased phagocytosis of macrophages to tumor cells. The immunosuppressive phenotype of TAMs is controlled by long-chain fatty acid metabolism (especially unsaturated fatty acids), which allows bone marrow-derived macrophages (BMDM) to polarize into M2 phenotypes with suppressive capacity; therefore, chemical inhibitors can effectively block TAM polarization *in vitro* and tumor growth *in vivo (* 100).

At the same time, the Exos from M1 macrophages (M1-Exos) have been proved to polarize macrophages into M1 phenotype (101–103). Recently, Wang et al. (104)showed that Thymosin α-1 (Tα-1) was internalized by TAMs by binding to phosphatidylserine (PtdSer) on the surface of apoptotic tumor cells and then combined with TLR7 and TLR8 on the lysosomal membrane to stimulate the downstream Myd88/SHIP1 and Myd88/IRAK4 signaling pathways, respectively, thereby reversing the M2 polarization of efferocytosis-activated macrophages and improving chemotherapeutic efficacy. In addition, Gunassekaran et al. (105)transfected M1 exosomes with NF-κB p50 siRNA and miR-511-3p to enhance M1 polarization and surface-modified M1 exosomes with IL4RPep-1, an IL4 receptor binding peptide, to target IL4R in TAMs (named IL4R-Exo (si/mi)). IL4R-Exo (si/mi) reprogrammed TAMs into M1 macrophages to inhibit tumor growth by downregulating target genes, reducing M2 markers and increasing M1 markers in M2 macrophages. This method may be a new approach for tumor immunotherapy. It is known that M2 polarization of macrophages is controlled by several transcription factors, such as signal transducer and activator of transcription 6 (STAT6), STAT3, and CCAAT/enhancer binding protein β (C/EBP β) (106). Therefore, Kamerkar et al. (107)designed and engineered exosome of antisense oligonucleotide (ASO) targeting STAT6 (exoASO-STAT6) and then transferred the exosome into TAMs. The results demonstrated that exoASO-STAT6 could induce the expression of M1 macrophage marker nitric oxide synthase 2 (NOS2), leading to TME remodeling, thereby reprogramming TAMs to a pro-inflammatory M1 phenotype and inducing CD8+ T cell-mediated adaptive immune responses.

### Targeting EMT

Due to the fact that EMT is a critical step in the process of tumor metastasis, targeting transcription factors of EMT or EMT-related pathways by miRNA and reversing the EMT process have been demonstrated to be effective strategies for the treatment of tumor metastasis. The miR-138 expression has been reported to be downregulated in a variety of cancers, such as colorectal cancer (108), melanoma (109), and ovarian cancer (110), suggesting that it is a tumor suppressor. Zhu et al. (87)showed that miR-138-5p inhibited EMT, growth, and metastasis of lung adenocarcinoma cells by targeting ZEB2. The transcription factor fork head box protein C2 (FOXC2) is required in the initiation and maintenance of EMT and in the acquisition of traits in CSCs, which enables cells to acquire higher motility, invasiveness, self-renewal, and drug resistance (111). One study reported that MC-1-F2, a small molecule inhibitor of FOXC2, reverses EMT and inhibits tumor metastasis by degrading FOXC2, blocking its nuclear localization, and inducing calmodulin conversion (88). As mentioned above, the TGF-β signaling pathway has a significant effect on EMT induction; for this reason, targeting this pathway is also an important means to prevent the occurrence of EMT. Glyunisertib (also known as LY2157299), an orally administered small molecule inhibitor of TGF-β receptor I kinase, specifically downregulates the phosphorylation of smad2, thereby inhibiting the activation of the typical TGF-β pathway (89). Also, TGF-β plays a vital role in embryonic development and normal cell physiology, making it a problematic anti-cancer therapeutic target. Liu et al. (90)have shown that targeting the EMT inducer CD146 by using engineered black phosphorus nanosheets (BPNSs) and mild photothermal treatment can convert highly metastatic mesenchymal breast cancer cells to an epithelial phenotype, while downregulating mesenchymal markers and upregulating epithelial markers, that is, the EMT in tumor cells is reversed, leading to tumor cell migration to be ceased entirely.

Besides, some drugs such as polyphenols, resveratrol, cellulose, lignans, and genistein flavonoids have also been proven to inhibit EMT and tumor progression (112). Zhang et al. (113)have studied that cellulose can enhance the radiosensitivity of SGC7901 cells and inhibit radiation-induced EMT and metastasis in both *in vitro* and nude mouse models, possibly due to inactivation of Notch-1 signaling and upregulation of miR-410. Lysine-specific histone demethylase 1 (LSD1), an epigenetic regulator that binds SNAIL and ZEB, demethylates H3K4 and H3K9. In many different types of cancer, LSD1 expression is associated with poor patient survival. LSD1 is recruited by EMT-TFs to their promoter regions, leading to epigenetic alterations that promote EMT (114). Several preclinical studies have shown that inhibition of LSD1 can effectively block EMT (91).

## Conclusion and future prospects

In this review, we briefly discuss the origin, classification, and potential mechanisms involved in tumor metastasis of TAMs, as well as the relevant molecular changes occurring in cells during EMT. We focus on the role of TAMs in promoting EMT in tumor cells and the current major anti-tumor therapies targeting TAMs and EMT.

With the further elucidation of the mechanisms of tumor metastasis, our understanding of the importance and molecular drivers of TAMs and EMT in cancer metastasis has been notably improved. The occurrence of tumor metastasis is influenced mainly by the various components of the tumor microenvironment (TME). TAMs, as the main components of TME, closely regulate tumor growth, immune escape, angiogenesis, and metastasis by secreting numerous factors. In recent years, targeting TAMs as a treatment strategy to prevent tumor progression and metastasis has drawn increasing attention from researchers. These strategies are based on TAMs depletion, inhibition of TAMs recruitment, and reprogramming TAMs. However, due to the significant heterogeneity of TAMs in regulating tumor metastasis; secondly, preclinical data obtained from the laboratory have not yielded satisfactory results when translated into clinical studies. Therefore, it is necessary to continue to explore more unknown mechanisms by which TAM promotes tumor metastasis. At the same time, because EMT gives tumor cells the ability to migrate and invade, therapeutic approaches that target the EMT process to stop tumor metastasis are currently under investigation. However, EMT also facilitates tissue development and wound healing under normal physiological conditions, so targeting the EMT process with anti-EMT drugs will inevitably bring some side effects to the organism. Therefore, it is essential to further in-depth studies on EMT and its molecular regulation during normal development, physiological state, and malignant transformation. Only then can potential therapeutic targets be identified to reduce possible side effects for patients.

## Author contributions

XL, XP, and XZ conceived the present study. XL and LC performed the literature search and data analysis. The first draft of the manuscript was written by XL and XP. XL, LC, XP, and XZ critically revised the work. All authors contributed to the article and approved the submitted version.

## Funding

This study was supported by grants from the National Natural Science Foundation of China (grant no. 82072707) and the Changhai Hospital 234 Project (no. 2020YXK029, 2019YXK019).

## Conflict of interest

The authors declare that the research was conducted in the absence of any commercial or financial relationships that could be construed as a potential conflict of interest.

## Publisher’s note

All claims expressed in this article are solely those of the authors and do not necessarily represent those of their affiliated organizations, or those of the publisher, the editors and the reviewers. Any product that may be evaluated in this article, or claim that may be made by its manufacturer, is not guaranteed or endorsed by the publisher.
